# Development of a Multiplex Real-Time PCR Assay for Mycobacterium bovis BCG and Validation in a Clinical Laboratory

**DOI:** 10.1128/Spectrum.01098-21

**Published:** 2021-09-08

**Authors:** Shannon C. Duffy, Manigandan Venkatesan, Shubhada Chothe, Indira Poojary, Valsan Philip Verghese, Vivek Kapur, Marcel A. Behr, Joy Sarojini Michael

**Affiliations:** a Department of Microbiology and Immunology, McGill University, Montreal, Quebec, Canada; b McGill International TB Centre, Montreal, Quebec, Canada; c Department of Clinical Microbiology, Christian Medical College Vellore, Vellore, Tamil Nadu, India; d Department of Animal Science, The Pennsylvania State University, University Park, Pennsylvania, USA; e Huck Institutes of the Life Sciences, The Pennsylvania State University, University Park, Pennsylvania, USA; f Department of Pediatric Infectious Diseases, Christian Medical College Vellore, Vellore, Tamil, India; g Department of Medicine, McGill University, Montreal, Quebec, Canada; Keck School of Medicine of the University of Southern California

**Keywords:** real-time PCR identification, BCG disease, disseminated BCG, *Mycobacterium bovis* BCG, *Mycobacterium tuberculosis*

## Abstract

Mycobacterium bovis bacillus Calmette-Guérin (BCG) is a live attenuated vaccine which can result in local or disseminated infection, most commonly in immunocompromised individuals. Differentiation of BCG from other members of the Mycobacterium tuberculosis complex (MTBC) is required to diagnose BCG disease, which requires specific management. Current methods for BCG diagnosis are based on mycobacterial culture and conventional PCR; the former is time-consuming and the latter often unavailable. Further, there are reports that certain BCG strains may be associated with a higher rate of adverse events. This study describes the development of a two-step multiplex real-time PCR assay which uses single nucleotide polymorphisms to detect BCG and identify early or late BCG strains. The assay has a limit of detection of 1 pg BCG boiled lysate DNA and was shown to detect BCG in both pure cultures and experimentally infected tissue. Its performance was assessed on 19 suspected BCG clinical isolates at Christian Medical College in Vellore, India, taken from January 2018 to August 2020. Of these 19 isolates, 10 were identified as BCG (6 early and 4 late strains), and 9 were identified as other MTBC members. Taken together, the results demonstrate the ability of this assay to identify and characterize BCG disease from cultures and infected tissue. The capacity to identify BCG may improve patient management, and the ability to discriminate between BCG strains may enable BCG vaccine pharmacovigilance.

**IMPORTANCE** Vaccination against tuberculosis with bacillus Calmette-Guérin (BCG) can lead to adverse events, including a rare but life-threatening complication of disseminated BCG. This complication often occurs in young children with immunodeficiencies and is associated with an ∼60% mortality rate. A rapid method of reliably identifying BCG infection is important because BCG requires treatment unique to tuberculosis. BCG is resistant to the first-line antituberculosis drug pyrazinamide. Additionally, diagnosis of BCG disease would lead to further investigation of a possible underlying immune condition. We have developed a diagnostic assay to identify BCG which improves upon previously published methods and can reliably identify BCG from bacterial culture or directly from infected tissue. This assay can also differentiate between strains of BCG, which have been suggested to be associated with different rates of adverse events. This assay was validated on 19 clinical isolates collected at Christian Medical College in Vellore, India.

## INTRODUCTION

Mycobacterium bovis bacillus Calmette-Guérin (BCG) is a live attenuated vaccine which is widely administered for protection against tuberculosis (1). BCG vaccination can be associated with local adverse events such as injection site abscesses or lymphadenitis. It may also lead to systemic infection, causing osteomyelitis or disseminated BCG (2). Disseminated disease is rare and often occurs in young children with primary immunodeficiency diseases (PIDs), such as severe combined immunodeficiency (SCID), chronic granulomatous disease (CGD), and Mendelian susceptibility to mycobacterial disease (MSMD) (3, 4). PIDs are a group of genetic disorders which cause impaired immunity and can result in increased susceptibility to infection and vaccine complications (4). Children with HIV are also at an increased risk of BCG complications (5). The estimated incidence of disseminated BCG is 0.06 to 3.4 cases per million, but nearly 1 per 100 in HIV-infected babies (3, 5, 6). Although the World Health Organization recommends against giving BCG to patients with documented immunodeficiency conditions, in India, as per the National Immunisation Programme, BCG is given soon after birth when these conditions have not yet been diagnosed (1, 2).

To diagnose BCG disease, laboratory differentiation of mycobacterial isolates is essential, as BCG has similar growth kinetics and morphology to M. tuberculosis strains. The treatment of patients with BCG infection will differ from those with tuberculosis since BCG is resistant to one of the first-line antituberculosis drugs, pyrazinamide (7). In addition, a diagnosis of BCG infection should lead to an immediate investigation into the patient’s immune status (4, 8). A BCG diagnosis would also confirm the absence of a contagion, and a public health inquiry into the source of infection would not need to be conducted. Current methods of diagnosis of BCG often rely on the detection of regions of difference (RD) by PCR (5, 9–13). RD-based approaches apply a rule-out strategy, where the detection of RDs is used to eliminate a potential mycobacterial species identity. A rule-in approach using lineage-defining single nucleotide polymorphisms (SNPs) may provide a simplified method of positively identifying mycobacteria. Additionally, conventional gel-based PCR is an open system which requires the running of a gel and is therefore not a pragmatic option in many clinical labs. This method can also only target one region of the genome at a time. Real-time PCR is advantageous because it is a closed system, requires a single step, and is easily multiplexed.

Given the increased data on M. tuberculosis SNPs and the availability of real-time PCR reagents that enable multiplexed analyses, we developed a two-step real-time PCR assay to rapidly identify BCG from a bacterial culture or directly from infected tissue. The two steps were designed to (i) identify BCG from other members of the MTBC and (ii) differentiate between early and late strains of BCG, something of interest in India, where two different BCG strains (BCG Russia and BCG Danish) with potentially different rates of adverse events are in use (2, 14–16). This assay was validated on 19 clinical isolates previously collected at Christian Medical College in Vellore, India, and our results suggest that the use of this assay may better inform patient management and enable BCG vaccine pharmacovigilance.

## RESULTS

### Specificity, reaction efficiency, and limit of detection.

**FIG 1:**
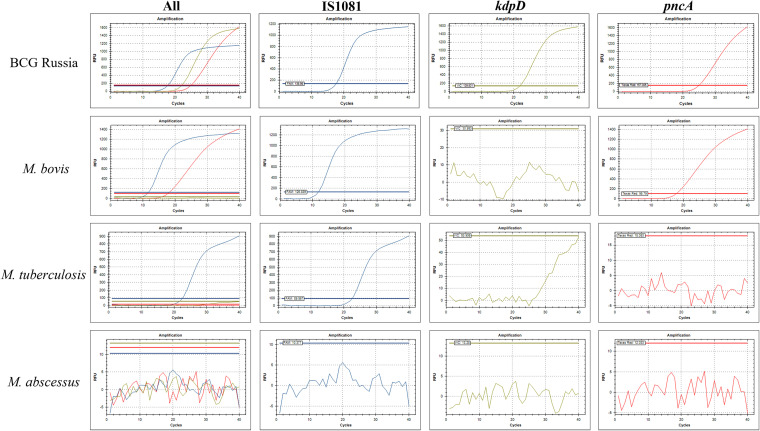
Step 1 assay specificity. A sample amplification curve of several mycobacteria with different probe amplification patterns using 10 ng of DNA: an MTBC subspecies M. tuberculosis, pyrazinamide-resistant M. bovis, a BCG strain BCG Russia, and a nontuberculous mycobacterium M. abscessus.

**FIG 2:**
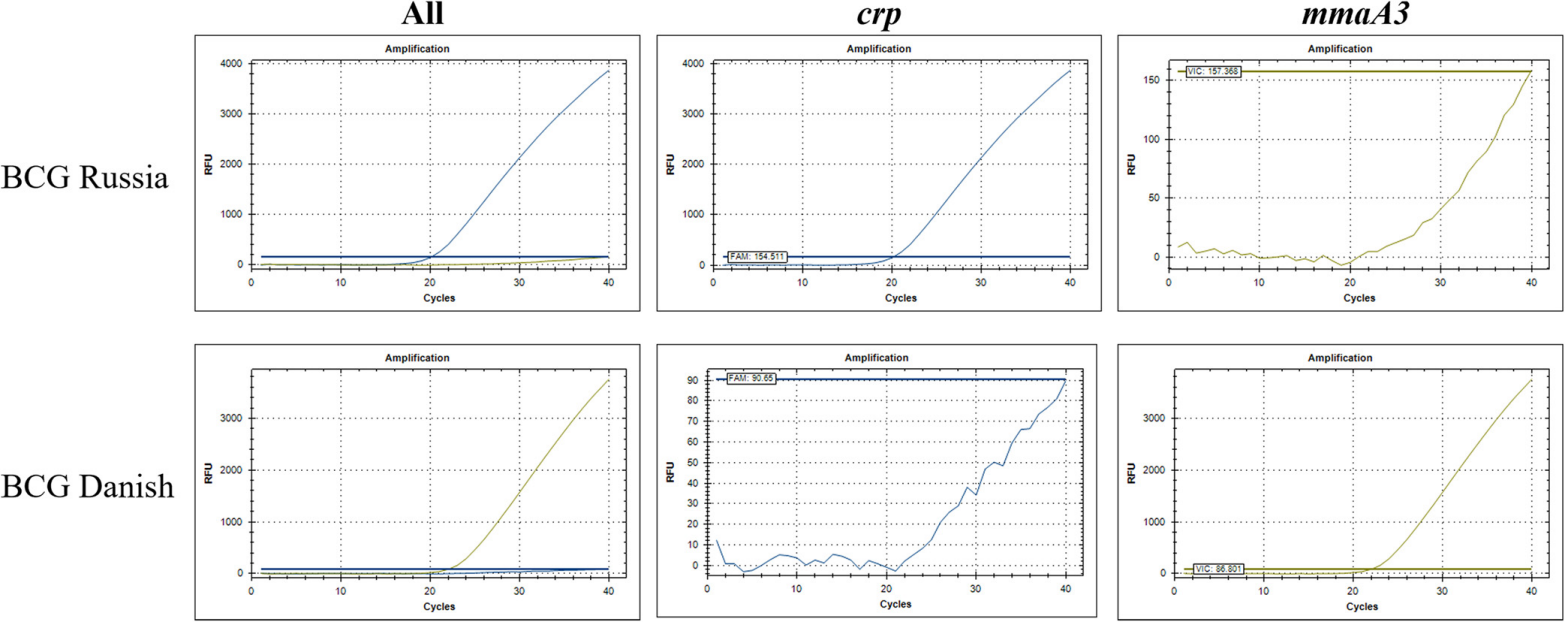
Step 2 assay specificity. A sample amplification curve for the early strain BCG Russia and the late strain BCG Danish using 10 ng DNA.

The specificity of the assay was tested on DNA from boiled lysate preparations of 26 isolates from 20 different mycobacterial species, including 8 BCG strains. The results for the step 1 and step 2 assays for all DNA correlated 100% as expected (Table S1 in the supplemental material). The expected step 1 amplification curves of several mycobacterial species are shown in Fig. 1. BCG was positive for all 3 probes (IS*1081*, *kdpD*, and *pncA*), M. bovis was positive for IS*1081* and *pncA*, M. tuberculosis was positive for IS*1081* only, and M. abscessus was negative for all 3 probes. The expected step 2 amplification curves of a BCG early and late strain are shown in Fig. 2. BCG Russia, an early strain, was positive for *crp*, and BCG Danish, a late strain, was positive for *mmaA3*. The reaction efficiencies of the probes were calculated by plotting the cycle threshold (*C_T_*) values from a serial dilution of BCG Russia boiled lysate DNA for step 1 (Fig. S1 in the supplemental material) and both BCG Russia and BCG Danish boiled lysate DNA for step 2 (Fig. S2 in the supplemental material). The reaction efficiencies for all probes ranged between 95.72 and 103.99%, which is within the range of desired values of 90 to 110% (Table 1). The limit of detection for step 1 was 1 pg of BCG Russia boiled lysate DNA. For step 2, the limit of detection was also 1 pg, whether using BCG Russia or BCG Danish boiled lysate DNA (Table 1). One picogram of BCG Russia DNA or 1 pg of BCG Danish DNA is estimated to equal 212.3 or 210 genome copies, respectively.

**TABLE 1:** Sequences, reaction efficiencies, and limits of detection of probes and primers

Assay	Name	Sequence (5′–3′)	Dye	Quencher	R^2^	Reaction efficiency (%)	Limit of detection (pg)
Step 1 (identify BCG)	IS1081_Probe	CTGAAGCCGACGCCCTGTGC	FAM	MGBNFQ	0.9971	95.72	1
	IS1081_Forward	GGCTGCTCTCGACGTTCATC					
	IS1081_Reverse	CGCTGATTGGACCGCTCAT					
							
	*kdpD*_Probe	ATGATCTCGCCGCGC	VIC	MGBNFQ	0.9997	103.99	1
	*kdpD*_Forward	GCAACAAGACCGCGAAACTG					
	*kdpD*_Reverse	GCAGTACTGCCTCCACATCGA					
							
	*pncA*_Probe	TGACGACTTCTCCGGCA	TEXAS RED	BHQ	0.9945	101.08	1
	*pncA*_Forward	AGCGGCGGACTACCATCAC					
	*pncA*_Reverse	TGTCCAGACTGGGATGGAAGT					
							
Step 2 (identify strain)	*crp*_Probe	TCGGCTGTACATCAT	FAM	MGBNFQ	0.9996	96.92	1
	*crp*_Forward	CCGTGGACACACGGTCTTC					
	*crp*_Reverse	GGCGACCGATCTTGACCTT					
							
	*mmaA3*_Probe	TCGTCGACTTGACC	VIC	MGBNFQ	0.9908	100.04	1
	*mmaA3*_Forward	CGAGCGCTACGACGTCAA					
	*mmaA3*_Reverse	CTGGCAGTAGGCGTGCTGAT					

### Assay reproducibility and robustness.

The inter- and intra-assay reproducibility was evaluated by comparing the *C_T_* values of 3 separate plates with 5 technical replicates per plate. The *C_T_* values remained consistent across all replicates: the *C_T_* values remained under 2 *C_T_* of difference across all replicates using step 1 (Fig. S3A in the supplemental material) and under 3 *C_T_* of difference across all replicates using step 2 (Fig. S3B in the supplemental material). The ability of all probes to produce the expected amplification plot in the presence of excess nonspecific DNA was tested using 1 ng target DNA (either BCG Russia or BCG Danish as indicated) with the addition of 10 ng of DNA from M. abscessus. All probes amplified as expected despite the presence of nonspecific DNA (Fig. S4 in the supplemental material).

### Identification from mice.

**FIG 3:**
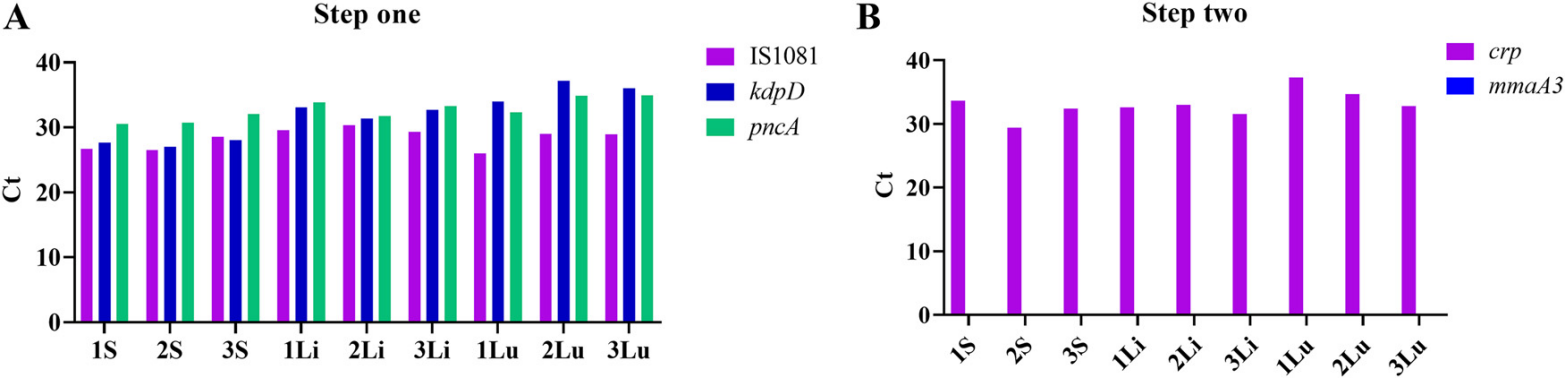
Real-time PCR results of DNA extracted directly from the tissue of BCG-infected mice. The *C_T_* values of DNA analyzed using the step 1 (A) and step 2 (B) assays. The number indicates the mouse, S indicates DNA extracted from the spleen, Li indicates DNA extracted from the liver, and Lu indicates DNA extracted from the lungs.

To determine whether the assay could be used to detect BCG directly from tissue, DNA was extracted from the spleen, liver, and lungs of 3 C57BL/6 mice which were intravenously infected with M. bovis BCG Russia. In all samples, the step 1 assay correctly identified the presence of BCG DNA (Fig. 3A), and the step 2 assay correctly identified the presence of an early BCG strain (Fig. 3B).

### Validation with clinical samples.

The assay was validated on a set of 19 isolates previously collected at Christian Medical College in Vellore, India (Table 2). Samples were taken from pus (6), lymph node (4), gastric aspirate (4), thigh sinus tract (1), bone (1), pleural tissue (1), colonic ulcer (1), and mastoid tissue (1). The samples came from patients in the following 5 states in India: Tamil Nadu (8), Andhra Pradesh (5), Karnataka (2), West Bengal (2), and Odisha (1). One sample was taken from a patient from Bangladesh. DNA was extracted from a culture on the mycobacteria growth indicator tube (MGIT) (13) or Lowenstein-Jensen (LJ) medium (5) or directly from a pus swab (1) (Table S2 in the supplemental material). Of the 19 isolates included, 10 (52.6%) were confirmed to be BCG. Four (40%) of the BCG isolates were from female patients. The median age of the BCG-infected patients was 6.5 months (range 3 to 12 months). In total, 4 of the BCG isolates came from patients diagnosed with immune conditions: 2 BCG isolates were from patients with SCID and 2 from those with MSMD (Table S2 in the supplemental material). Of the 10 BCG isolates, 6 (60%) were identified as a BCG early strain, and 4 (40%) were identified as a BCG late strain. Sample amplification plots of 2 clinical isolates, 1 early strain and 1 late strain, are provided in Fig. S5 in the supplemental material. The other 9 isolates included in this study were identified as MTBC by the step 1 assay. Two (∼22.2%) of the MTBC isolates came from female patient samples. Of the 9 MTBC isolates, 7 (∼77.8%) came from patients between 1 and 2 years old (the maximum age of inclusion).

**TABLE 2:** Patient information and assay results of 19 suspected BCG clinical isolates

Isolate no.	Age (mo)	Sex	Location	Sample type	Assay result
1	3	M	Karnataka	Abscess pus	BCG late strain
2	3	F	Andhra Pradesh	Gastric aspirate	MTBC
3	4	M	Karnataka	Lymph node pus	BCG late strain
4	5	F	Tamil Nadu	Lymph node	BCG early strain
5	5	F	Tamil Nadu	Lymph node pus	BCG late strain
6	6	M	Tamil Nadu	Lymph node	MTBC
7	6	M	Andhra Pradesh	Lymph node	BCG early strain
8	7	M	Andhra Pradesh	Thigh sinus tract biopsy specimen	BCG early strain
9	9	F	Tamil Nadu	Lymph node	BCG early strain
10	9	F	Tamil Nadu	Lymph node pus	BCG late strain
11	10	M	West Bengal	Talar bone	BCG early strain
12	12	M	Tamil Nadu	BCG site pus	BCG early strain
13	12	F	Odisha	Gastric aspirate	MTBC
14	12	F	Tamil Nadu	Lymph node	MTBC
15	18	M	Andhra Pradesh	Gastric aspirate	MTBC
16	19	M	Andhra Pradesh	Pleural tissue	MTBC
17	20	M	Bangladesh	Colonic ulcer biopsy specimen	MTBC
18	22	M	West Bengal	Gastric aspirate	MTBC
19	24	M	Tamil Nadu	Mastoid tissue	MTBC

## DISCUSSION

A rapid method of differentiating BCG from other mycobacterial species is essential to providing optimal care for BCG infection, particularly in cases of disseminated BCG, which is associated with a mortality rate of ∼60% (6, 8, 17–20). A method of determining whether an early or late strain is causing a case of disseminated BCG may also provide an opportunity for pharmacosurveillance, to determine whether certain strains are linked to a higher rate of adverse events (2, 14). Here, we have developed a two-step multiplex real-time PCR assay to rapidly identify BCG isolates from other mycobacterial species and differentiate between BCG early and late strains. This assay was shown to detect BCG from a culture, directly from the tissue of infected mice, or from a patient pus swab in a case where a culture was unavailable. The ability to identify BCG directly from a clinical sample circumvents time-consuming mycobacterial culture to inform patient treatment. This assay was tested on a small validation set of 19 clinical isolates previously collected at Christian Medical College in Vellore, India, where two different BCG strains are in use: BCG Russia (an early strain) and BCG Danish (a late strain). There was no assay for BCG detection in use at Christian Medical College prior to conducting this study. If clinicians suspected a patient was infected with BCG based on their age, disease presentation, and immune status, they were treated accordingly. Additionally, we are not aware of any real-time PCR assays which detect BCG using SNPs, nor are we aware of any which can discriminate between BCG strains. Of the 19 isolates tested, 10 were identified as BCG, 6 of which were early strains.

The clinical management of a patient diagnosed with BCG disease will differ compared to a patient with M. tuberculosis. Unlike M. tuberculosis, BCG infection would not trigger the need for contact tracing to identify the source of infection and perhaps other infected persons. Confirmation of BCG infection may also affect which drugs would be included in the patient treatment regimen. Since BCG is derived from M. bovis, all strains are naturally resistant to pyrazinamide, a first-line antituberculosis drug (7). The assay described in this study confirms pyrazinamide resistance using the *pncA* probe. In addition, the *mmaA3* probe used to identify late strains detects a SNP which is associated with a loss of methoxymycolate production and is suggested to cause low levels of isoniazid resistance in late strains (7, 21). A diagnosis of BCG infection should also lead to further investigation of possible underlying immune conditions, which may not have been recognized at the time of disease presentation (4, 8). In a previous study of 349 SCID patients, 118 developed disseminated BCG disease, a 33,000-fold increase over the general population (17). In this study, of the 10 isolates identified as BCG, 2 came from patients confirmed to have SCID and 2 came from patients confirmed to have MSMD (Table S2 in the supplemental material). Although BCG vaccination is contraindicated in people with these conditions, the Universal Immunisation Programme (UIP) recommends BCG vaccination in India at birth or as early as possible (https://www.nhp.gov.in/universal-immunisation-programme_pg), so these rare immune conditions are usually not recognized until after vaccination.

There are 14 different BCG strains, which have evolved with considerable genetic and phenotypic heterogeneity among them. BCG strains differ from one another by genetic deletions, SNPs, and duplications (15). It has been suggested that some BCG strains may be associated with a higher rate of adverse events (2, 14–16). This possibility is of particular interest in India, where both an early and a late BCG strain are currently in use. In Finland, BCG osteitis rates were as high as 36.9 per 100,000 while using the early strain BCG Sweden. Upon switching to the late strain BCG Glaxo in 1978, that rate decreased to 6.4 per 100,000 (2, 14). Conversely, in phase 1 of a randomized control trial in which BCG Danish was compared with BCG Russia, lymphadenitis was reported in 6 out of 582 patients given BCG Danish and 1 out of 575 patients given BCG Russia (16). In this study, 6 out of 10 BCG isolates were early strains, presumably BCG Russia. The other 4 were identified as late strains, presumably BCG Danish. The sample size of this study is too small to draw conclusions concerning an association between the BCG strain and adverse events; however, screening is ongoing, and this question may be revisited with the established assay as the total sample size rises.

To conclude, we have developed a rapid method of reliably identifying BCG early and late strains which improves upon previously described assays. This assay may be used to inform patient treatment without the time constraints of mycobacterial culture. The ability to differentiate between early and late strains may also inform BCG vaccine pharmacovigilance in a country in which two vaccine strains are currently in use.

## MATERIALS AND METHODS

### Assay design.

A two-step real-time PCR protocol was designed to identify BCG isolates from clinical samples. Step 1 identifies BCG from other MTBC subspecies. It is a three-probe multiplex real-time PCR assay which detects the MTBC insertion element IS*1081*, the BCG-specific SNP *kdpD* c247t, and the pyrazinamide resistance SNP *pncA* c169g (7, 22). Step 2 differentiates BCG isolates as early or late strains. It is a two-probe real-time PCR assay which detects the BCG early strain SNP in *crp* c140t and the BCG late strain SNP in *mmaA3* g293a (7). The probes and primers were designed using Primer Express v. 3.0 (Applied Biosystems, Foster City, CA, USA). All primer and probe sequences are listed in Table 1. The IS*1081*, *kdpD*, *crp*, and *mmaA3* probes are TaqMan MGB probes (Applied Biosystems), and the *pncA* probe is a PrimeTime BHQ probe (Integrated DNA Technologies, Coralville, IA, USA). All primers were purchased from Invitrogen (Carlsbad, CA, USA).

### Real-time PCR.

All real-time PCRs were performed in a 20-μl reaction mixture containing 1 μl DNA at a concentration of 10 ng/μl unless otherwise specified. Step 1 reaction mixtures were prepared with 10 μl TaqMan multiplex master mix (Applied Biosystems), 100 nM IS*1081* forward and reverse primers, 300 nM forward and reverse *kdpD* primers, 900 nM *pncA* forward and reverse primers, 125 nM IS*1081* probe, 125 nM *kdpD* probe, and 500 nM *pncA* probe. Step 2 real-time PCR mixtures were prepared using 10 μl TaqMan multiplex master mix, 300 nM *crp* and *mmaA3* forward and reverse primers, 250 nM *crp* and *mmaA3* probes, and 1 μl dimethyl sulfoxide (Sigma-Aldrich, St. Louis, MO, USA). Real-time PCRs were performed using a CFX96 touch real-time PCR detection system (Bio-Rad, Hercules, CA, USA) under the following thermocycling conditions: one cycle at 95°C for 10 min followed by 40 cycles of 95°C for 15 s and 63°C for 1 min. A summary of how results are interpreted is found in Fig. S6 in the supplemental material.

### Specificity.

The specificity of the two-step assay was evaluated with the following bacterial species: M. tuberculosis, M. bovis, M. orygis, M. africanum, M. caprae (2), M. microti, M. avium subsp. *paratuberculosis* (4), M. avium subsp. *avium* (3), M. avium subsp. *hominissuis*, M. kansasii, M. abscessus, M. smegmatis, BCG Russia, BCG Moreau, BCG Sweden, BCG Japan, BCG Danish, BCG Pasteur, BCG Prague, and BCG Glaxo. If more than one isolate of each mycobacterium was tested, the number of isolates is indicated in parentheses. Cultures of each bacterial species were grown in 7H9 Middlebrook medium (Becton, Dickinson, Franklin Lakes, NJ, USA), ADC (Becton, Dickinson), 0.2% glycerol (Sigma-Aldrich), and 0.05% Tween 80 (Sigma-Aldrich) to approximately an optical density at 600 nm (OD_600_) of 0.5 to 0.8. The medium was supplemented with 1 μg/ml ferric mycobactin J (Allied Monitor, Fayette, MO, USA) for M. avium subsp. *paratuberculosis* cultures. One milliliter of culture was added to a screw cap microcentrifuge tube and centrifuged for 5 min at 13,000 rpm. The supernatant was discarded, and the pellet was resuspended in 200 μl molecular grade water. The tube was boiled at 95°C for 40 min and then centrifuged again for 5 min at 13,000 rpm. The supernatant was transferred to a new tube for real-time PCR use. Boiled lysate DNA was used in this study to demonstrate the assay robustness and evaluate the assay using a DNA preparation that would be a pragmatic and convenient option for many labs. The prepared DNA was stored at −20°C until ready for testing by real-time PCR.

### Reaction efficiencies, limits of detection, reproducibility, and robustness.

The reaction efficiencies of all probes were determined by plotting the *C_T_* values obtained from a standard curve of 10-fold dilutions of DNA isolated from BCG Russia or BCG Danish. The reaction efficiency was calculated using the formula *E* = (10^−1/^*^m^* − 1) × 100%, where *m* is the slope of the line. The limit of detection of all probes was determined by the minimum amount of DNA to yield a positive amplification result from the above standard curves. The inter- and intra-assay reproducibility was evaluated by comparing the *C_T_* values of the step 1 and step 2 assays completed on 3 separate plates with 5 technical replicates per plate. The ability of the assay to function in the presence of excess nonspecific DNA was tested by performing real-time PCR with 1 ng of target DNA (BCG Russia or BCG Danish) and 10 ng of M. abscessus DNA.

### Mouse infection.

The ability of the assay to identify BCG directly from tissue was determined using infected mice. Three C57BL/6 mice (Jackson Laboratories, Bar Harbor, MA, USA) were intravenously infected with 2.3 × 10^8^ CFU BCG Russia. After 3 weeks, the mice were sacrificed, and spleen, liver, and lung samples were collected. The organs were homogenized, and infection was confirmed by serial dilution and plating onto Middlebrook 7H10 agar (Becton, Dickinson), OADC enrichment (Becton, Dickinson), 0.5% glycerol (Sigma-Aldrich), and PANTA antibiotic mixture (Becton, Dickinson). DNA was extracted from organ homogenates as previously described (23). DNA was then quantified and analyzed by the two-step real-time PCR assay.

### Clinical samples.

The assay was validated using 19 clinical isolates previously collected at Christian Medical College, Vellore, India, from January 2018 to August 2020. This study obtained approval and ethical clearance from the Institutional Review Board (IRB) of Christian Medical College, Vellore (IRB approval number 11725, dated 19 December 2018). An isolate is defined as a pure microbe cultured from an individual patient. A sample is defined as the patient specimen provided for culture. Isolates were collected from patients presenting with suspected tuberculosis who were 2 years of age or younger or by request of the clinician due to suspected BCG disease. Clinicians suspected BCG involvement with a BCG site abscess or axillary lymphadenitis, or when disseminated MTBC was isolated in an infant with an immunodeficiency disorder such as SCID or MSMD. DNA was extracted from positive mycobacteria growth indicator tube (MGIT) or Lowenstein-Jensen (LJ) cultures as available. In one sample, a culture was not available, and DNA was extracted directly from a pus swab. DNA was prepared by boiling for samples collected before March 2019 or by using the QIAamp DNA minikit (Qiagen, Hilden, Germany) for samples collected after March 2019. All DNA was analyzed by the step 1 real-time PCR assay. If an isolate was identified as BCG in step 1, it was analyzed by step 2.
