# Supplementary material for: Development of a Multiplex Real-Time PCR Assay for Mycobacterium bovis BCG and Validation in a Clinical Laboratory
Source: Microbiol Spectr. 2021 Sep 8;9(2):e01098-21. doi: 10.1128/Spectrum.01098-21 (PMC8557883; doi:10.1128/Spectrum.01098-21)
Supplement: SUPPLEMENTAL FILE 1 — Supplemental material. Download Spectrum.01098-21-s0001.pdf, PDF file, 0.5 MB [file spectrum.01098-21-s0001.pdf]

Table S1: Summary of specificity results from 26 isolates

Isolate	IS1081	*kdpD*	*pncA*	*crp*	*mmaA3*
M. tuberculosis H37Rv	+	-	-		
M. bovis AF2122/97	+	-	+		
M. orygis 51145	+	-	-		
M. africanum MT18 7400	+	-	-		
M. caprae 6032	+	-	-		
M. caprae 65749	+	-	-		
M. microti	+	-	-		
M. abscessus ATCC 19977	-	-	-		
MAP K10^{+}	-	-	-		
MAP Holland	-	-	-		
MAP 6758	-	-	-		
MAP 8645	-	-	-		
MAH 104	-	-	-		
MAA 15769	-	-	-		
MAA 35713	-	-	-		
MAA 35718	-	-	-		
M. kansasii ATCC 12478	-	-	-		
M. smegmatis MC^{2}155	-	-	-		
BCG Russia	+	+	+	+	-
BCG Danish	+	+	+	-	+
BCG Pasteur	+	+	+	-	+
BCG Sweden	+	+	+	+	-
BCG Moreau	+	+	+	+	-
BCG Glaxo	+	+	+	-	+
BCG Japan	+	+	+	+	-
BCG Prague	+	+	+	-	+

Boxes filled in gray indicate not applicable

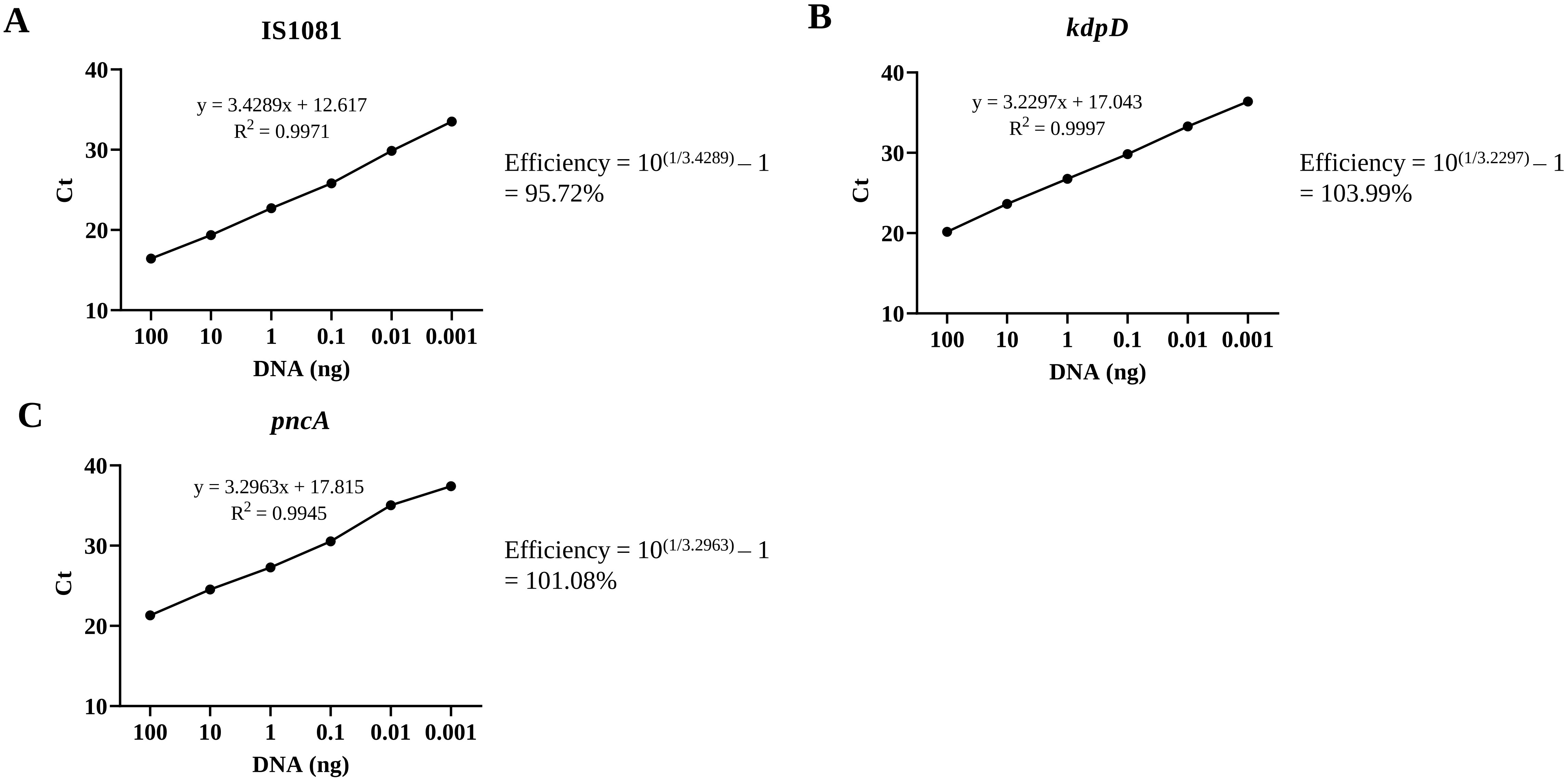

Figure S1: Reaction efficiency calculations of step one probes
(A-C) A plot of the Ct values of a 10-fold serial dilution of BCG Russia DNA and calculation of the reaction efficiency for the IS1081 probe (A), the *kdpD* probe (B) and the *pncA* probe (C).

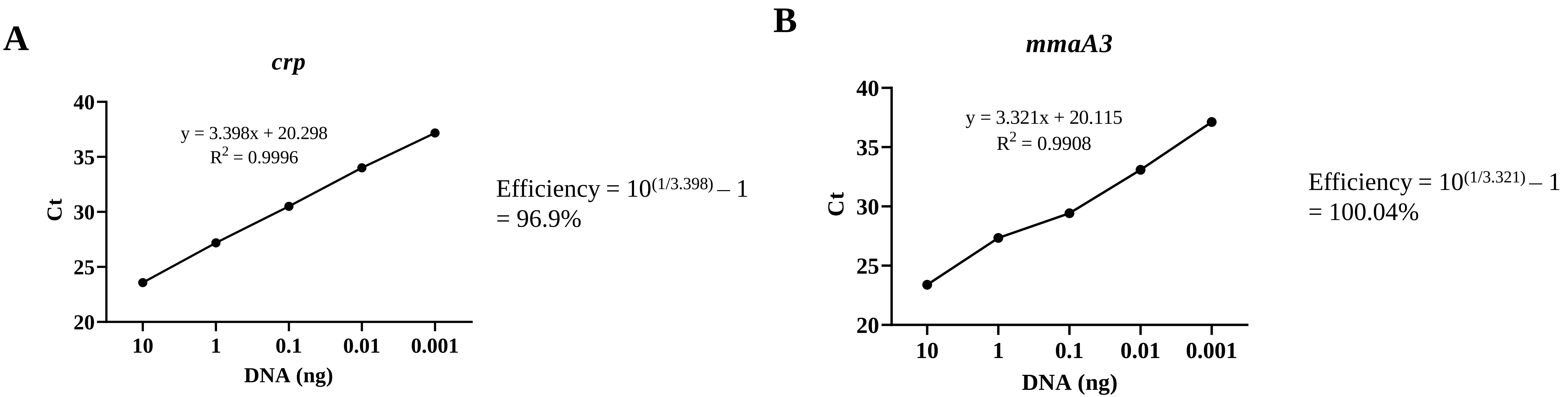

Figure S2: Reaction efficiency calculations of step two probes

(A) A plot of the Ct values of a 10-fold serial dilution of BCG Russia DNA and calculation of the reaction efficiency of the *crp* probe. (B) A plot of the Ct values of a 10-fold serial dilution of BCG Danish DNA and calculation of the reaction efficiency of the *mmaA3* probe.

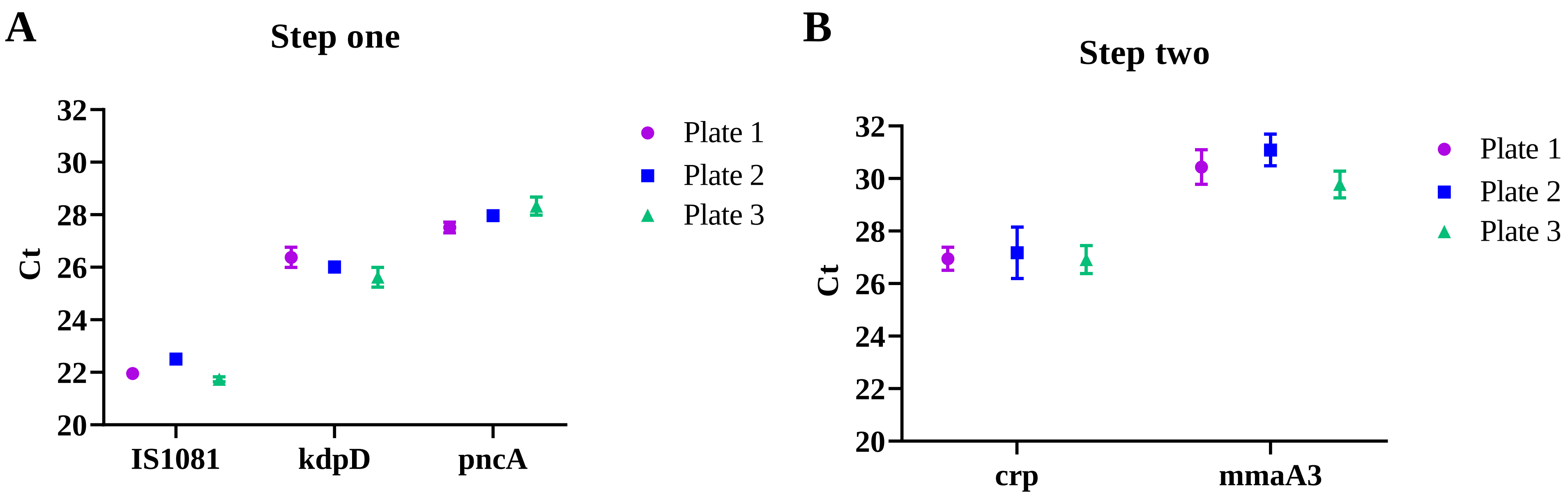

Figure S3: Inter- and Intra-assay reproducibility
(A, B) The Ct values were compared using 1 ng of BCG Russia DNA with step 1 (A) or step 2 (B) assay. The inter-assay reproducibility was evaluated by comparing the Ct values across five technical replicates. The intra-assay reproducibility was evaluated by comparing the Ct values across three assay replicates.

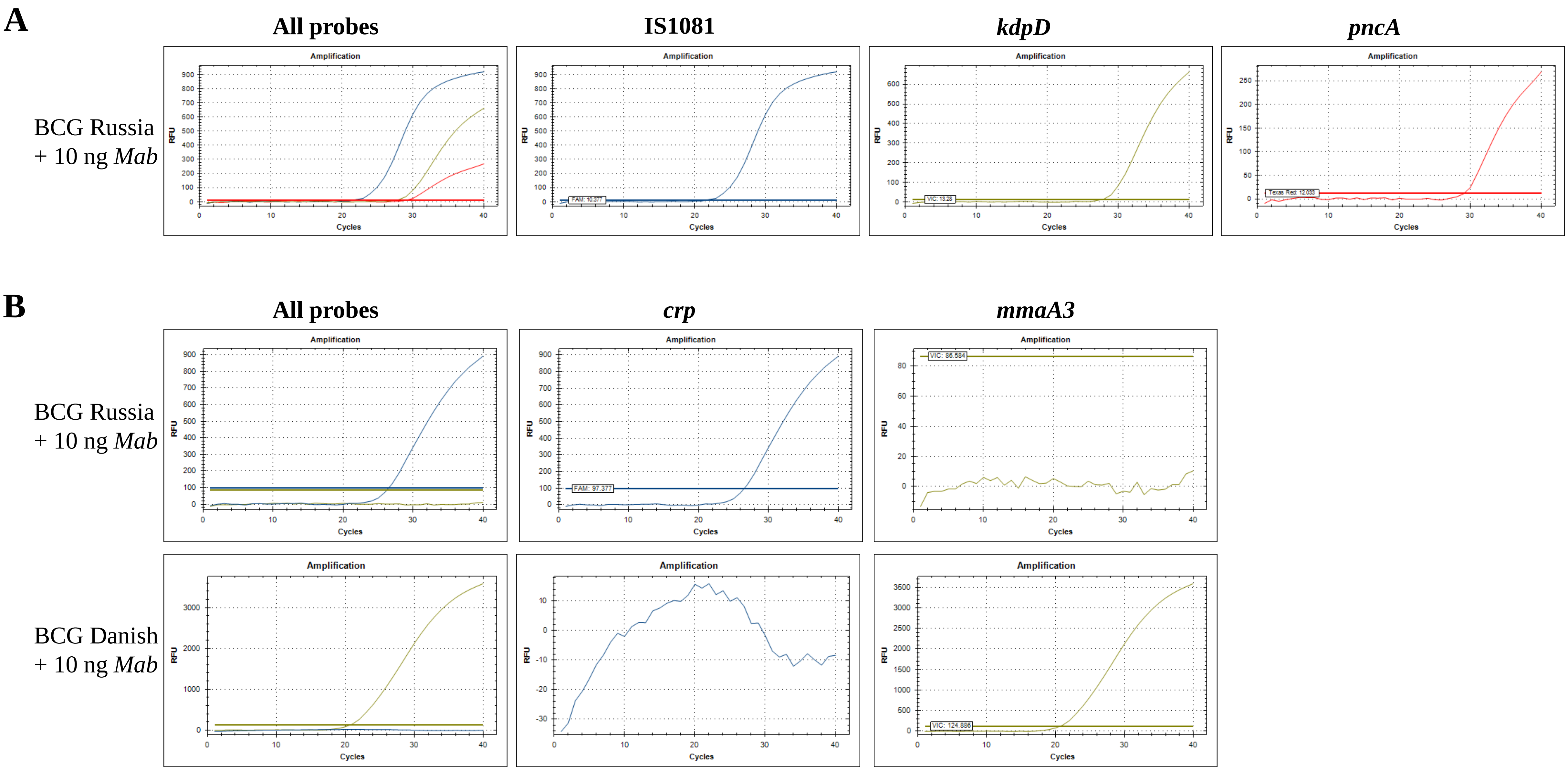

Figure S4: Assay performance in presence of 10ng of excess non-specific DNA

The ability of the assay to produce the expected amplification plot in the presence of excess non-specific DNA was evaluated. (A) A total of 10 ng of DNA from *M. abscessus* (*Mab*) was added to a step one reaction mixture containing 1 ng of BCG Russia DNA. (B**)** A total of 10 ng of DNA from *Mab* was added to a step one reaction mixture containing 1 ng of either BCG Russia or BCG Danish DNA.

Table S2: Clinical and DNA extraction information for 19 suspected BCG clinical isolates

Isolate No.	Age (months)	Sex	Location	Sample type	Clinical disease	Year of diagnosis	Extraction from	Extraction method	Assay result
1	3	M	Karnataka	Abscess pus	Failure to thrive; BCG abscess	2020	LJ	Qiagen kit	BCG Late strain
2	3	F	Andhra Pradesh	Gastric aspirate	TB meningitis and PTB	2020	MGIT	Qiagen kit	MTBC
3	4	M	Karnataka	Lymph node pus	Axillary lymphadenitis	2019	LJ	Qiagen kit	BCG Late strain
4	5	F	Tamil Nadu	Lymph node	Axillary lymphadenitis	2018	MGIT	Heat prep	BCG Early strain
5	5	F	Tamil Nadu	Lymph node pus	Axillary lymphadenitis	2020	LJ	Qiagen kit	BCG Late strain
6	6	M	Tamil Nadu	Lymph node	Submandibular lymphadenitis	2019	MGIT	Heat prep	MTBC
7	6	M	Andhra Pradesh	Lymph node	MSMD, disseminated BCG infection	2018	MGIT	Heat prep	BCG Early strain
8	7	M	Andhra Pradesh	Thigh sinus tract biopsy	Post-immunization abscess with sinus tract on thigh	2020	LJ	Qiagen kit	BCG Early strain
9	9	F	Tamil Nadu	Lymph node pus	SCID, PCP, failure to thrive, disseminated BCG infection	2019	MGIT	Heat prep	BCG Early strain
10	9	F	Tamil Nadu	Lymph node pus	MSMD, axillary lymphadenitis	2020	Pus swab	Qiagen kit	BCG Late strain
11	10	M	West Bengal	Talar bone	Ankle tubercular arthritis	2019	MGIT	Qiagen kit	BCG Early strain
12	12	M	Tamil Nadu	BCG site pus	SCID, BCG site ulceration	2018	MGIT	Heat prep	BCG Early strain
13	12	F	Odisha	Gastric aspirate	Congenital TB	2020	MGIT	Qiagen kit	MTBC
14	12	F	Tamil Nadu	Lymph node	TB cervical lymphadenopathy	2020	MGIT	Qiagen kit	MTBC
15	18	M	Andhra Pradesh	Gastric aspirate	PTB	2019	MGIT	Heat prep	MTBC
16	19	M	Andra Pradesh	Pleural tissue	TB pleural effusion	2019	MGIT	Heat prep	MTBC
17	20	M	Bangladesh	Colonic ulcer biopsy	Chronic active colitis	2019	MGIT	Heat prep	MTBC
18	22	M	West Bengal	Gastric aspirate	TB meningitis and PTB	2019	MGIT	Qiagen kit	MTBC
19	24	M	Tamil Nadu	Mastoid tissue	Tubercular mastoiditis	2020	LJ	Qiagen kit	MTBC

PTB – pulmonary tuberculosis, MSMD – Mendelian susceptibility to mycobacterial disease, SCID – Severe combined immunodeficiency disorder, PCP – Pneumocystis pneumonia, CNS – central nervous system, MGIT – mycobacterial growth indicator tube, LJ – Lowenstein Jensen medium, MTBC – *Mycobacterium tuberculosis* complex

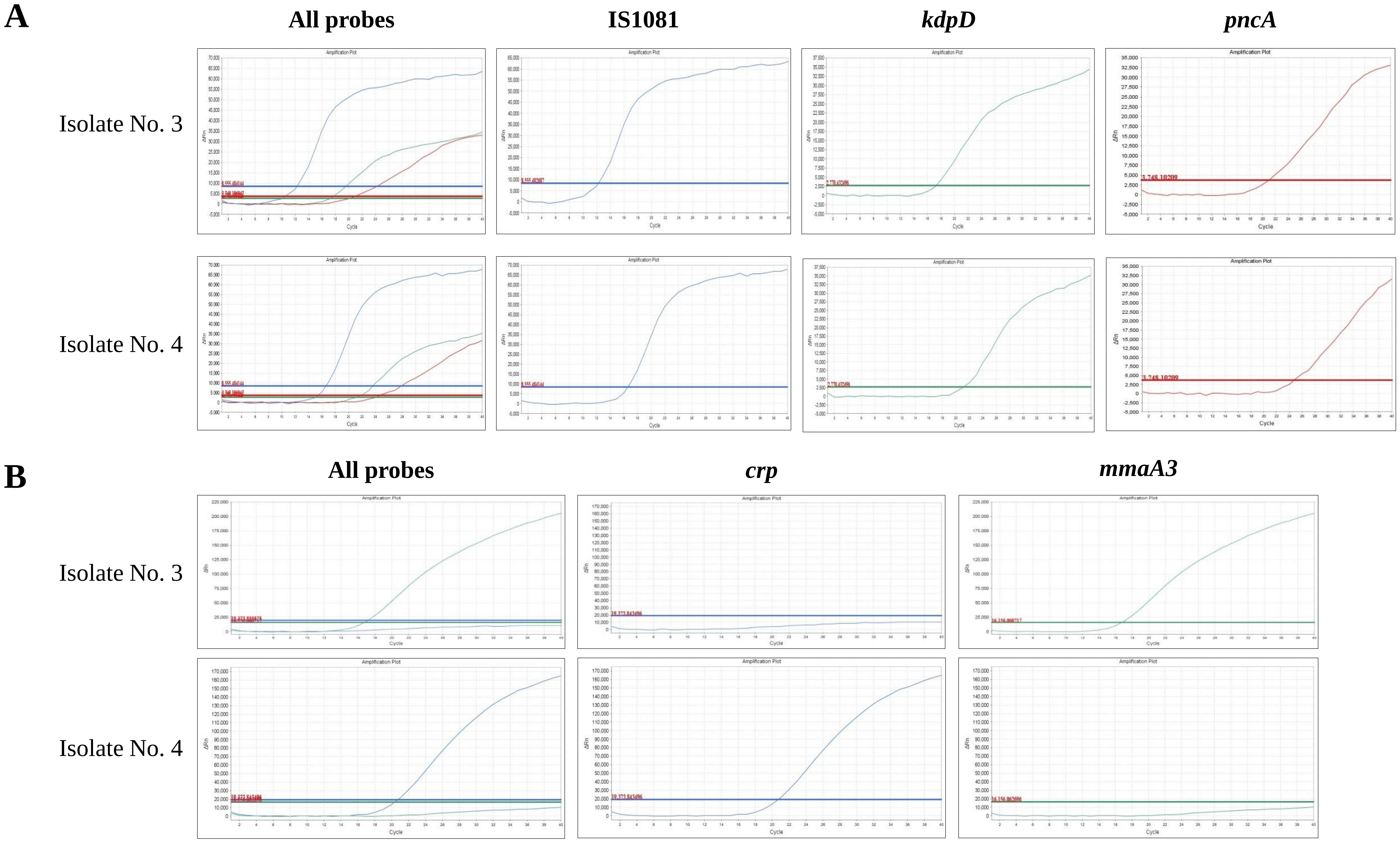

Figure S5: Clinical isolates amplification plots

(A, B) Example amplification plots of two clinical isolates using the step one (A) and step two (B) assay. Isolate number 3 was identified as a BCG late strain and isolate number 4 was identified as a BCG early strain.

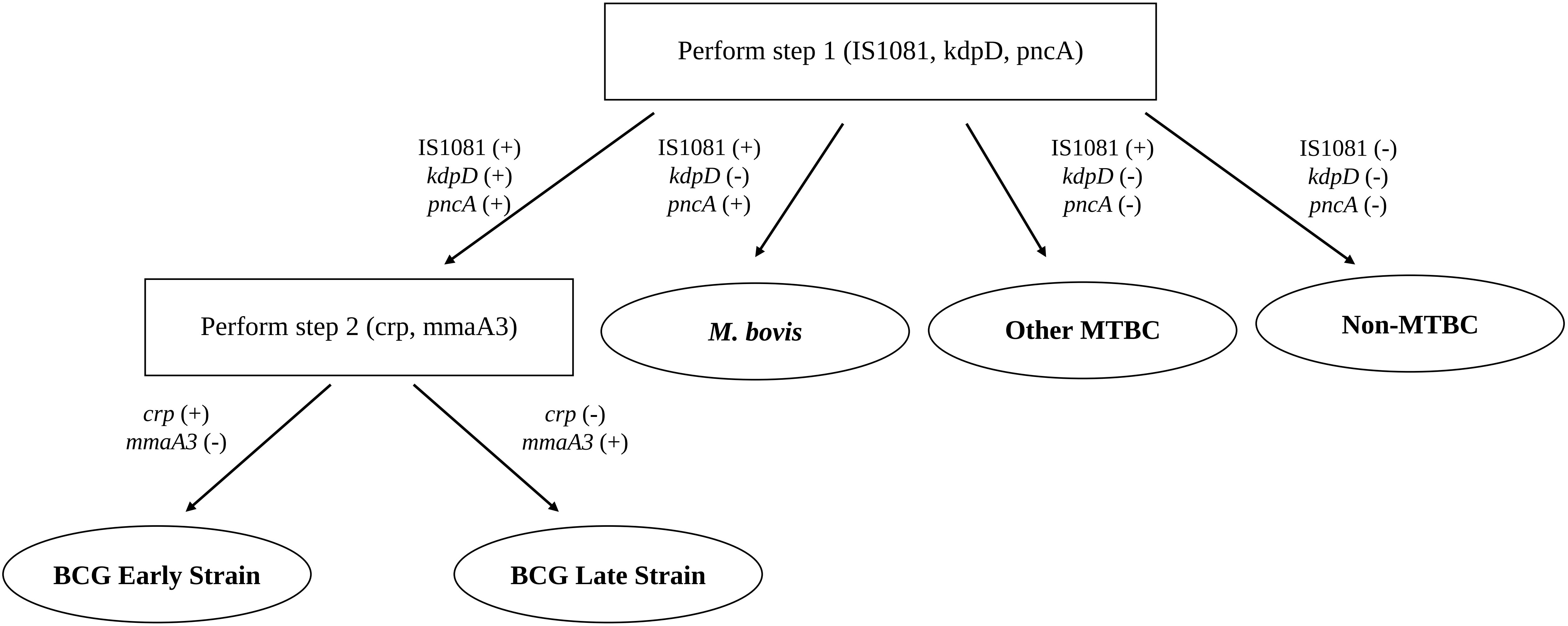

Figure S6: Assay workflow and interpretation
